# 3-dimensional analysis of hard- and soft-tissue symmetry in a Chinese population

**DOI:** 10.1186/s12903-023-03163-z

**Published:** 2023-06-29

**Authors:** Jiamin Zhao, Yifei Xu, Jinxiu Wang, Zhen Lu, Kun Qi

**Affiliations:** 1 Key Laboratory of Shaanxi Province for Craniofacial Precision Medicine Research, College of Stomatology, Xi’an Jiaotong University, 98 XiWu Road, 710004 Xi’an, Shaanxi P.R. China; 2 Department of Orthodontics, Stomatological Hospital of Xi’an Jiaotong University, 98 XiWu Road, 710004 Xi’an, Shaanxi P.R. China; 3 Department of Oral Anatomy and Physiology and TMD, School of Stomatology, The Fourth Military Medical University, Xi’an, China

**Keywords:** Facial asymmetry, Orthodontics, Computer-assisted image analysis, Orthognathic surgery, Cone-Beam CT

## Abstract

**Background:**

Facial symmetry severely affects appearance and function. Large numbers of patients seek orthodontic treatment to improve facial symmetry. However, the correlation between hard- and soft-tissue symmetry is still unclear. Our aim was to investigate the hard- and soft-tissue symmetry in subjects with different levels of menton deviation and sagittal skeletal classes with 3D digital analysis and to investigate the relationship between the entire and individual hard- and soft-tissues.

**Methods:**

A total of 270 adults (135 males and 135 females) consisting of 45 subjects of each sex in each sagittal skeletal classification group. All subjects were further classified into relative symmetry (RS), moderate asymmetry (MA) and severe asymmetry (SA) groups based on the degree of menton deviation from the mid-sagittal plane (MSP). The 3D images were segmented into anatomical structures and mirrored across the MSP after establishing a coordinate system. Original and mirrored images were registered by a best-fit algorithm, and the corresponding root mean square (RMS) values and colormap were obtained. The Mann‒Whitney U test and Spearman correlation were conducted for statistical analysis.

**Results:**

The RMS increased with greater deviations with regard to the deviation of the menton in most of anatomical structures. Asymmetry was represented in the same way regardless of sagittal skeletal pattern. The soft-tissue asymmetry had a significant correlation with dentition in the RS group (0.409), while in the SA group, it was related to the ramus (0.526) and corpus (0.417) in males and was related to the ramus in the MA (0.332) and SA (0.359) groups in females.

**Conclusions:**

The mirroring method combining CBCT and 3dMD provides a new approach for symmetry analysis. Asymmetry might not be influenced by sagittal skeletal patterns. Soft-tissue asymmetry might be reduced by improving the dentition in individuals with RS group, while among those with MA or SA, whose menton deviation was larger than 2 mm, orthognathic treatment should be considered.

**Supplementary Information:**

The online version contains supplementary material available at 10.1186/s12903-023-03163-z.

## Background

Facial symmetry is an ideal condition in which the shape, size and distance to an arbitrary reference plane are similar on the right and left sides [1]. In contrast, asymmetry is a common phenomenon found in both hard and soft tissues [2]. The incidence of facial asymmetry ranges from 11–37% worldwide [3]. This condition can also compromise attractiveness, functional activities and physical health. There is no absolute facial symmetry; indeed, there is a normal range in which facial asymmetry is considered acceptable. Most patients seek orthodontic and orthopedic treatment to address complaints of facial asymmetry [4]. To develop an effective treatment plan and estimate the corresponding curative effect on the patient’s facial asymmetry, it would be worthwhile to calculate the deviation of hard- and soft-tissue anatomical structures as well as the association between them in different degrees of asymmetry.

Facial asymmetry has been analyzed using manual and automatic 2D and 3D techniques. Symmetry analysis using 2D images has several limitations, such as a lack of sufficient anatomical landmarks and sensitivity to the shooting angle and head position [5, 6]. Facial asymmetry is determined in multiple directions; therefore, 3D evaluation was eventually deemed necessary [7]. Three-dimensional techniques, such as computed tomography (CT), cone beam CT (CBCT), facial casting and laser scanning, are currently used to assess facial asymmetry [8]. Traditional anthropometric methods are mainly based on several asymmetry indices, such as symmetry rate [9], deviation of linear measurements [6, 10, 11], distance to the reference plane [12] and Euclidean distance [13]. Compared to manual methods, digital methods have been confirmed to be more accurate due to the comprehensiveness of the associated analysis and the lower technical error of measurement [14]. Among these computer-aided methodologies, registration of the original and mirrored images with deviations measured is a common approach that has been employed in several studies [1, 5, 14]. However, these studies mostly focused on specific hard structures, Caucasian populations, and limited asymmetrical classifications, and few have investigated hard and soft tissue at the same time.

Sagittal skeletal patterns are also essential factors in orthodontic diagnosis and treatment planning. The relationship between sagittal skeletal patterns and facial asymmetry is still unclear. Some scholars believe that asymmetry is much more prevalent in Class III subjects due to jaw displacement caused by posterior crossbite [15]. However, other studies found that the asymmetry was distributed equally among Class I, II and III malocclusions [16]. Epidemiological investigations may be affected by factors such as ethnicity and research method. To further analyze the morphological differences among various sagittal skeletal classes, some studies have made comparisons between Classes I and III [17], Classes I and II [18], and Classes II and III [19]. Previous studies typically considered Class I symmetry subjects as the control group. However, few have analyzed all the sagittal skeletal types with different menton deviations.

The present article aimed to describe the hard- and soft-tissue asymmetry in patients with different levels of menton deviation from the midsagittal plane (MSP) and different sagittal skeletal classes and to investigate the relationship between the entire and individual hard- and soft-tissues. Our study consisted of digital analyses combining CBCT techniques for hard-tissue and noncontact, precise 3D facial photographs (3dMD images) for soft-tissue, whose results are important for the diagnosis and treatment planning of facial asymmetry.

## Materials and methods

Hard- and soft-tissue of 270 subjects with varying degrees of facial asymmetry and sagittal classification was segmented into several anatomical regions, including zygomatic process, maxilla, ramus, nose, cheek and so forth using CBCT and 3dMD imaging techniques. After establishing a coordinate system, the entire images and segmentations were mirrored across the mid-sagittal plane. The original and mirrored shells of hard- and soft-tissue were superimposed with the RMS values and colormap obtained. Statistical analyses were conducted to investigate the relationship between soft- and hard-tissue asymmetry as well as the influence of gender and sagittal skeletal classification on symmetry.

### Radiographical images and 3D photographs

A total of 270 subjects between 18 and 30 years of age (135 males and 135 females) were selected for our study. All of the subjects were randomly selected from the Stomatology Hospital of Xi’an Jiaotong University, Xi’an, Shaanxi, China. Exclusion criteria included previous orthodontic or orthognathic treatment, facial trauma, cleft palates, metal prostheses or condylar pathology. Inclusion criteria included a lack of history of plastic surgery and a body mass index between 18.5 and 24.9. Patients who met the inclusion criteria were then classified into groups according to the deviation of the hard tissue menton point from MSP and sagittal skeletal classes. According to the former, following the scheme established by Zheng et al. [20], the patients were classified into the relative symmetry (RS) group if the deviation was no larger than 2 mm, the moderate asymmetry (MA) group if the deviation was larger than 2 mm but no more than 4 mm, and the severe asymmetry (SA) group if the deviation was larger than 4 mm according to the previous studies [21, 22]. Based on the sagittal skeletal patterns which were determined by lateral cephalometric images derived from CBCT images, the subjects were classified into skeletal Class I (2° ≤ ANB angle ≤ 5°), Class II (ANB angle > 5°), and Class III (ANB angle < 2°), in which the ANB angle describes the relative position between the maxilla and mandible as described by Ricketts [23]. In total, 135 subjects were evenly distributed in Classes I, II and III, with 45 subjects in each group. Furthermore, for each sagittal skeletal pattern, 15 male and female RS, MA and SA patients were also identified. Informed consent was obtained from all participants. The sample size that gave a two-armed 95% power at a two-sided 1% level of significance was calculated using PASS software (Power Analysis and Sample Size; NCSS, Kaysville, Utah, USA) based on Duran et al. [1]. For each subject, CBCT (KaVo 3D eXam i, Germany, with the parameters: 230 V, 5 A, 50/60 Hz, and 1,150 VA) and 3dMD (3dMD, Atlanta, GA, USA) images [24] were obtained by experienced doctors for diagnostic reason like malocclusion.

### Extraction and registration of hard and soft tissue

**Fig. 1:**
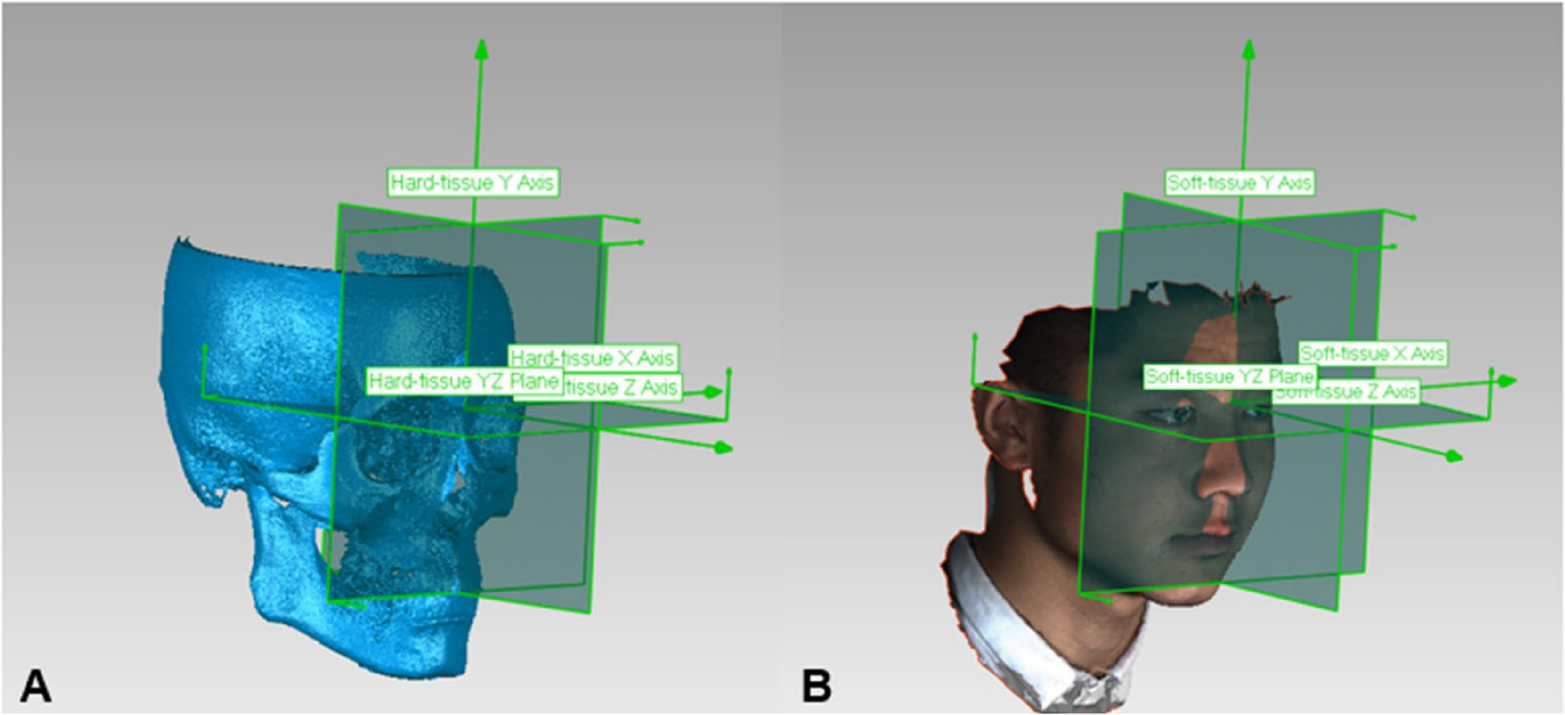
The three-dimensional coordinate system of hard-tissue (**A**) and soft-tissue (**B**). For the hard-tissue, the Y-Z plane (MSP) was set across the nasion point and perpendicular to the frontozygomatic suture line. The X-Z plane (horizontal reference plane) was set through the nasion point and parallel to Frankfurt horizontal plane which was set across the left orbitale point and bilateral porion points. The X-Y plane (coronal reference plane) was set through the nasion point and perpendicular to the above two planes. For the soft-tissue, The Y-Z plane (MSP) was set through the soft tissue nasion point and perpendicular to the line connecting the exocanthion points on the right and left sides. The X-Z plane (horizontal reference plane) was set through the nasion point and parallel to Frankfurt horizontal plane. The X-Y plane (coronal reference plane) was also set through the nasion point and perpendicular to the above two planes.

First, CBCT data were imported into MIMICS (Materialise’s interactive medical image control system) V21.0 (Materialise, Switzerland), where the hard tissue was extracted with a threshold ranging from 226 to 3071 Hounsfield units (HU) meanwhile region growing was conducted to minimize the amount of noise. Second, the hard tissue images were stored in standardized stereolithography (STL) file format and transferred into Geomagic Wrap (Raindrop Geomagic Inc, Research Triangle Park, NC, USA). Third, the position of the hard tissue images was corrected, and a coordinate system was established (Fig. 1A). The relative landmarks are shown in Table 1.

**Table 1:** Abbreviation and definition of hard- and soft-tissue landmarks

	Landmarks	Abbreviation	Definition
Hard-tissue	Frontozygomatic suture	FZ	Medial point on the orbital rim of the zygomaticofrontal suture
	Nasion	N	Middle point of the nasofrontal suture
	Zygion	Zy	Middle point of the zigomaticotemporal suture
	Porion	Po	Most upper point of meatus acusticus externus
	Anterior point	Ap	Deepest point of ramus anterior border
	Antegonial notch	An	Deepest point of the loncavity between ramus and corpus of the mandible
	Menton	Me	The most inferior midpoint on the symphysis
	Orbitale	Or	Most inferior point of the infraorbital margin
Soft-tissue	Exocanthion	Ex	Point at the outer commissure of the eye fissure
	Soft tissue orbitale	Or'	Most inferior point of infraorbital rim
	Soft tissue nasion	N'	Most retruded point in the tissue overlying area of the frontonasal suture
	Subnasale	Sn	Midpoint on the nasolabial soft tissue contour between the columella crest and the upper lip
	Cheilion	Ch	Point located at each labial commissure
	Tragus	Tra	Prominence in front of the opening of the outer ear canal
	Labiale inferius	Li	Midline soft tissue point directly overlying the hard tissue menton
	Menton'	Me'	The most inferior midpoint on the symphysis in the soft tidssue
	Cervical point	Cr	Intersection point of neck and chin region

**Fig. 2:**
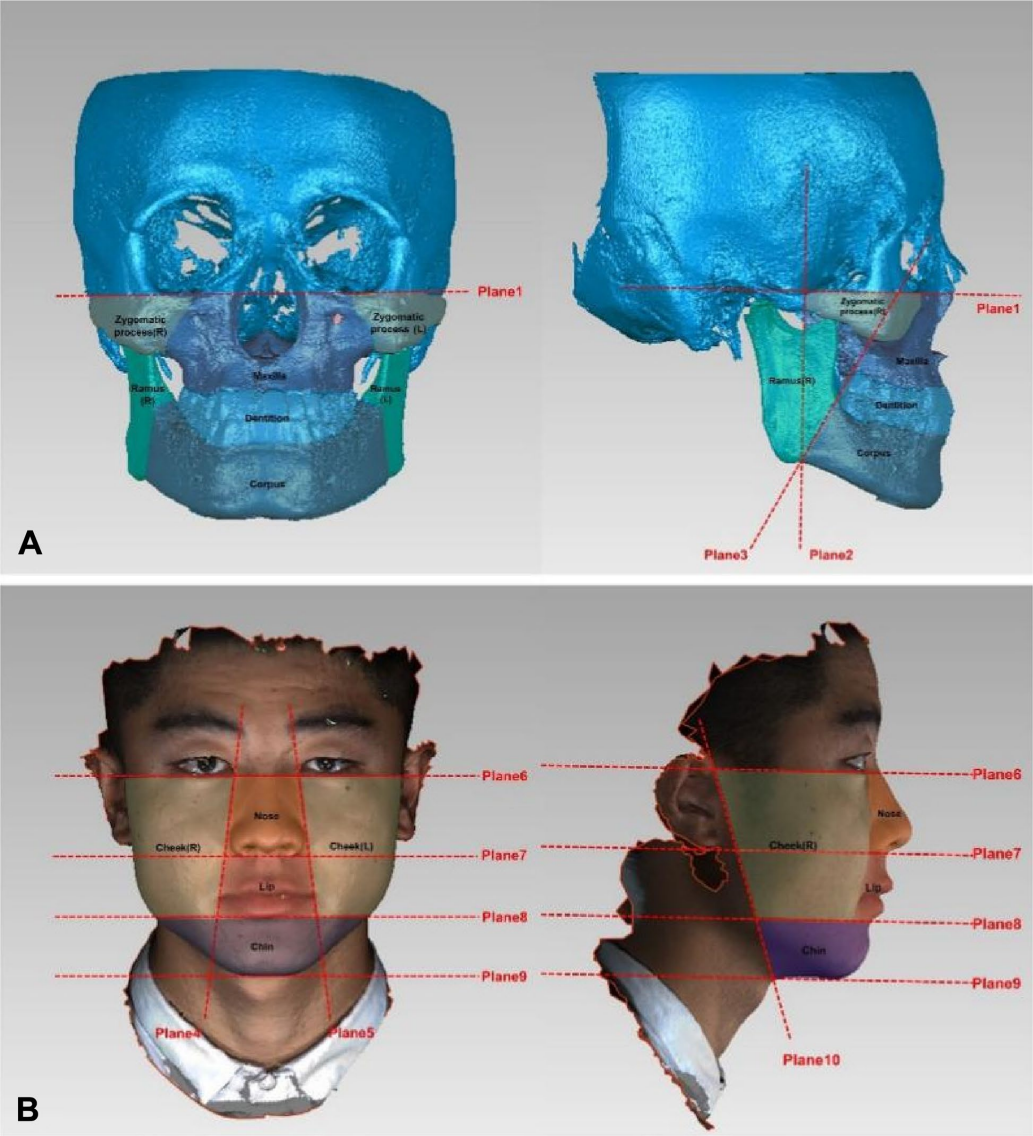
5 Segmented structures of hard-tissue (**A**) and 4 soft-tissue (**B**) in frontal and lateral views with the planes 1,4,5,6,7,8 and 9 perpendicular to the coronal reference plane while planes 2,3 and 10 are perpendicular to the horizontal reference plane in the hard- or soft- tissue coordinate system: Plane1OrL-OrR; Plane2 Through Zy and perpendicular to the Plane 1; Plane3 Ap-An; Plane4 ExR-ChR; Plane5 ExL-ChL; Plane6 Or’L-Or’R; Plane7 Through Sn and parallel to the X-Z plane; Plane8 Through Li and parallel to the X-Z plane; Plane9 Through Me’ and parallel to the X-Z plane; Plane10 Cr-Tra.

**Fig. 3:**
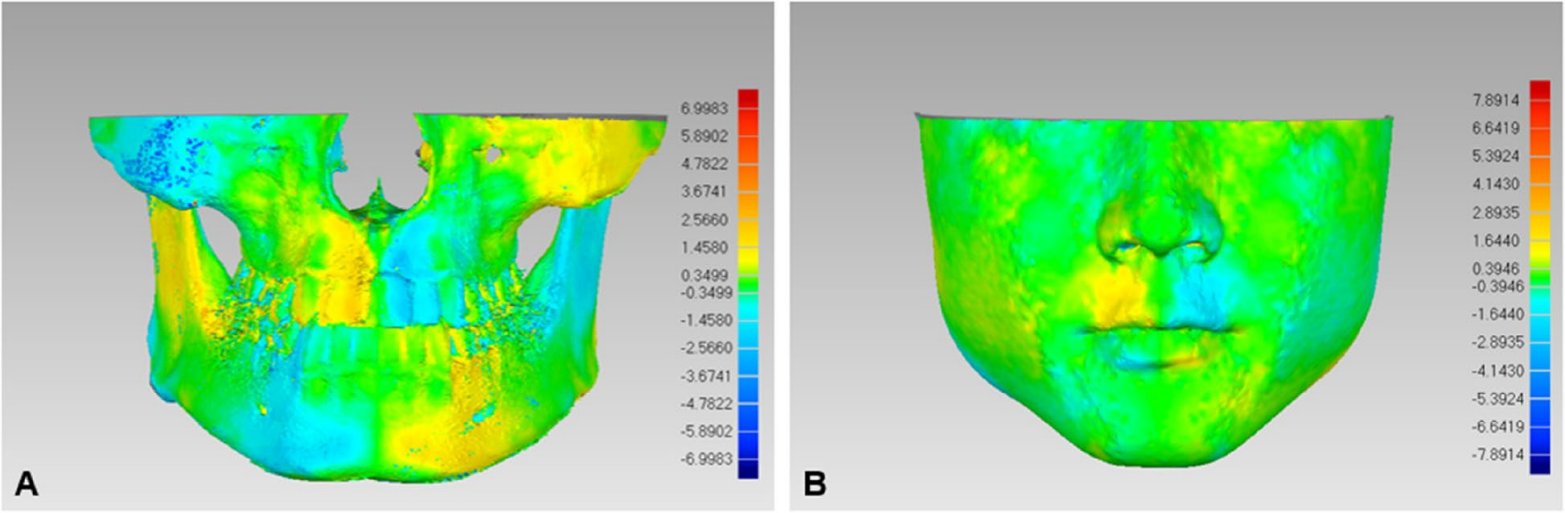
Color map obtained by the registration of the original and mirrored images. **A** hard-tissue; **B** soft-tissue.

After establishment of the coordinate system, the cranial structure was removed through planes 1 and 2 (Fig. 2A). The extracted hard tissue images were then separated into 5 anatomical structures, including the zygomatic process, maxilla, dentition, ramus of the mandible and corpus of the mandible, according to a previous study [1] (Fig. 2A). The entire hard tissue and 5 anatomical structures were mirrored through the MSP. Finally, the original images and mirrored images were registered with superimpositions performed separately for the entire body and each structure by the best-fit algorithm in Geomagic Wrap, and the root mean square (RMS) distances were measured to calculate the deviation and used to generate a colormap to show the deviation in every region (Fig. 3A).

Similar to the hard tissue, the soft tissue images were also stored in STL format and transferred into Geomagic Wrap. After correcting for head position, the coordinate system was established using the corresponding soft tissue landmarks similar to the corresponding hard-tissue (Fig. 1B). In addition, the confounding regions such as hair, eyes, ears and neck were removed using the plane 6 and 10 (Fig. 2B). The extracted soft tissue was then separated into four anatomical regions, including the cheek, nose, lip and chin, according to a previous study [25] (Fig. 2B). The entire soft tissue and the 4 anatomical regions were mirrored through the MSP. Finally, the original images and the mirrored images were overlapped separately, and the RMS values and colormap were obtained (Fig. 3B).

### Test of reproducibility of the procedure

To assess the reproducibility of the procedure, 10 males and 10 females were randomly selected from the 270 subjects. Then, the hard and soft tissue was extracted again twice at 14-day intervals after the first assessment by the same and an additional operator, and the RMS was measured again. The inter- and intraclass coefficients were calculated.

### Statistical analysis

Statistical analysis was conducted by using Statistical Product and Service Solutions (SPSS) software, version 18.0, for Windows. The Kolmogorov–Smirnov test revealed that the distribution of the RMS values in the hard and soft tissues was nonnormal with z values of 1.757 (*p* = 0.004*) in hard tissue and 2.413 (*p* = 0.000*) in soft tissue. Thus, the Mann‒Whitney U test was applied to analyze the difference in asymmetry between males and females as well as among the RS, MA and SA groups and among Classes I, II and III. The Spearman correlation coefficient was calculated to assess the association between hard and soft tissue. The level of significance was defined as *p* < 0.05.

## Results

### Consistency and distribution results

The inter- and intraclass coefficients were 0.916 (*p* = 0.000*) and 0.945 (*p* = 0.000*), respectively, showing that the extraction and registration of the hard- and soft-tissue were reproducible and responsible. The Kolmogorov‒Smirnov test showed that the distributions of the hard- and soft-tissue RMS values were nonnormal, with z values of 1.757 (*p* = 0.004*) in hard tissue and 2.413 (*p* = 0.000*) in soft tissue. Consequently, subsequent statistical analyses were conducted using non-parametric tests.

### Asymmetry of hard- and soft-tissue in males and females

The RMS in most regions exhibited significant sexual dimorphism, with larger RMS values in males, including the entire hard- and soft-tissue, maxilla, dentition, corpus and nose suggesting that males exhibited greater asymmetry than females (Table 2). The zygomatic process demonstrated the highest RMS value with a mean of 1.192 mm in males and 1.145 mm in females while the nose showed the lowest RMS value with a mean of 0.468 mm in males and 0.365 mm in females. Additionally, there was a significant difference in the RMS values of hard- and soft-tissue between males (*p* = 0.000*) and females (*p* = 0.000*), with the hard-tissue exhibiting larger values.

**Table 2:** RMS (mm) point-to-point distances in hard and soft tissue in males and females

	Males				Females					
	Min	Max	Mean	Median	Min	Max	Mean	Median	Z	*P*
Hard Tissue	0.860	2.570	1.308	1.263	0.813	2.653	1.213	1.153	-3.436	0.001^*^
Soft Tissue	0.454	2.250	1.053	0.910	0.414	2.358	0.915	0.802	-2.644	0.008^*^
Zygomatic Process	0.578	2.207	1.192	1.149	0.560	2.049	1.145	1.095	-0.920	0.357
Maxilla	0.632	1.621	1.097	1.082	0.717	1.423	0.993	0.971	-5.280	0.000^*^
Dentition	0.594	1.575	1.081	1.059	0.551	1.677	1.000	0.970	-3.322	0.001^*^
Ramus	0.607	2.344	1.034	0.963	0.577	2.372	0.984	0.912	-1.594	0.111
Corpus	0.566	1.795	0.898	0.881	0.505	1.899	0.818	0.778	-3.721	0.000^*^
Nose	0.216	0.947	0.468	0.443	0.192	0.808	0.365	0.357	-6.968	0.000^*^
Cheek	0.266	2.377	1.007	0.869	0.312	2.570	0.912	0.821	-1.686	0.092
Lip	0.162	2.021	0.540	0.467	0.190	1.067	0.477	0.435	-1.708	0.088
Chin	0.221	2.100	0.827	0.785	0.266	1.906	0.730	0.647	-1.913	0.056

*Statistically significant, *p* < 0.05

### Asymmetry of hard- and soft-tissue in each group based on the deviation of menton

**Fig. 4:**
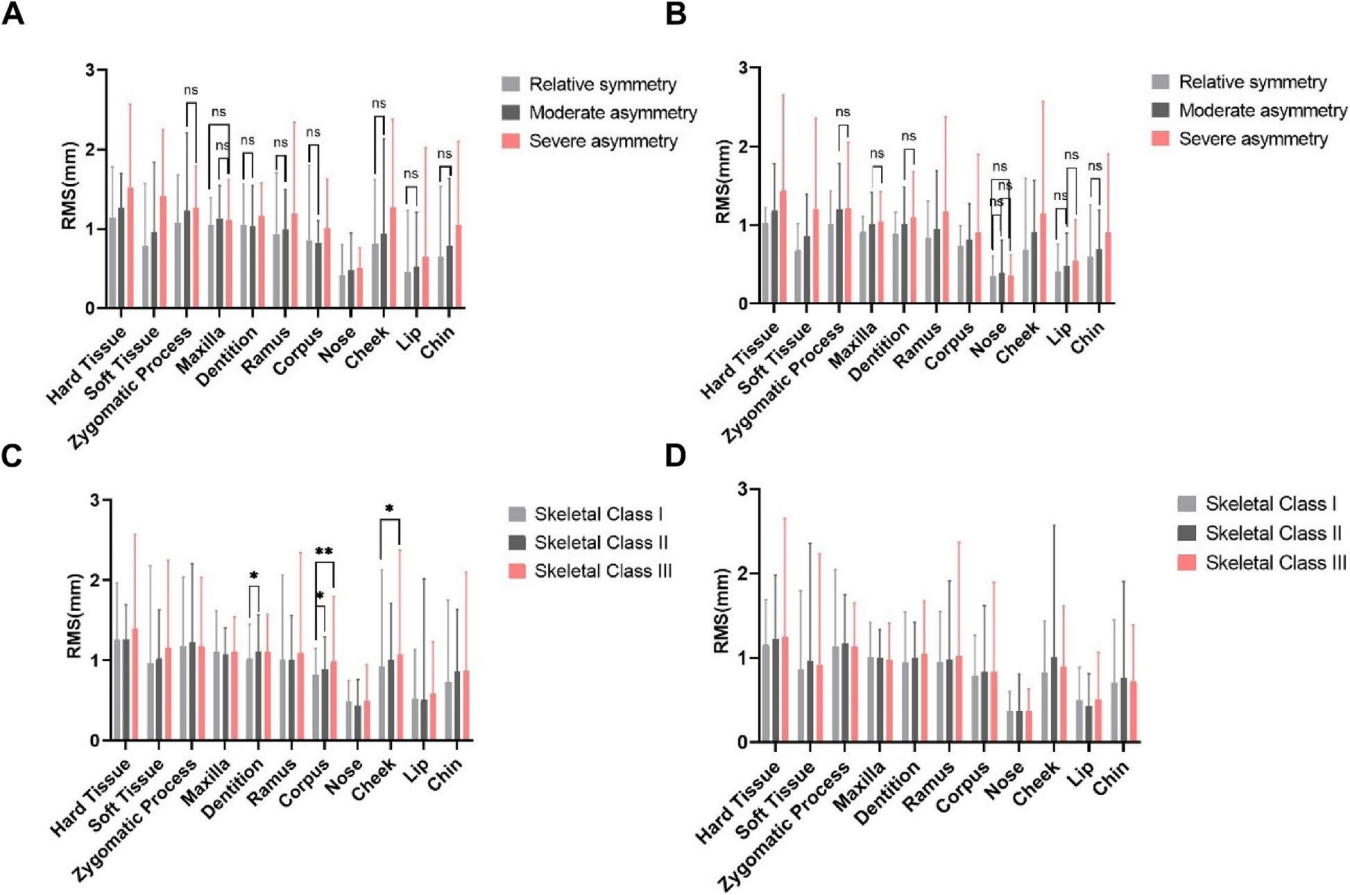
The mean and range of the RMS expressing the asymmetry in hard- and soft-tissue in each group based on the deviation of menton (**A**, **B**) and sagittal skeletal classes (**C**, **D**). **A** (in males) and **B** (in females) showed the RMS in groups with different deviation of menton where “ns” represented no significance was found between two groups. Except for these groups showed no significant difference, others exhibited significant difference with *p*-value lower than 0.05. **C** (in males) and **D** (in females) showed the RMS in groups with different sagittal skeletal classes where “*” represented significance difference was found with *p*-value lower than 0.05 and “**” represented *p*-value was lower than 0.01. Others showed no significant difference.

With regard to the deviation of the menton, the RMS increased with greater deviations in both males and females (Fig. 4A B, Supplementary Table s1-4). Significant differences in RMS values of entire hard- and soft-tissue were observed among RS, MA and SA groups in both sexes. In males, significant differences were found between RS and SA groups in most regions except for hard tissue maxilla. No difference was found between RS and MA group in the dentition, ramus, corpus, cheek, lip and chin (Fig. 4A). In females, significant differences were found among the RS, SA and MA groups in terms of ramus, corpus and cheek features. However, no differences were detected in soft tissue features especially in nose (Fig. 4B).

### Asymmetry of hard- and soft-tissue in each group based on sagittal skeletal classes

Sagittal skeletal classes showed no significant differences in most regions in males and in all regions in females (Fig. 4C D, Supplementary Tables 5–8). In males, Class II had a higher dentition RMS than Class I, with mean values of 1.113 mm and 1.021 mm, respectively. In the corpus region, the RMS was higher in Class III and II, with a mean value of 0.985 mm and 0.892mm, than in Class I, with a mean value of 0.817 mm. However, no difference was found between the RMS of Class II and Class III. In the soft tissue, the RMS of the cheek was higher in Class III than in Class I, with means of 1.092 and 0.928 mm, respectively.

### Correlation of asymmetry of hard- and soft-tissue

Both the entire and individual hard- and soft-tissue showed moderate to high correlations in their RMS values (Supplementary Tables 9 and 10). In males, the RMS value of the entire hard-tissue was highly correlated with those of the maxilla, ramus, and corpus. The highest correlation was observed between the RMS values of the entire hard-tissue and those of the ramus. The RMS value of the entire soft tissue showed moderate to high correlations with those of the cheek and chin, while lower correlations were found for nose and lip. It is worth mentioning that the cheek RMS exhibited a moderate correlation with the dentition RMS in the RS group, whereas no significant correlation was found in the MA and SA groups. Similar trends were observed for both soft-tissue and lip RMS, while the ramus and corpus demonstrated a significant correlation with entire soft-tissue solely in the SA group. In females, similarly, the entire soft-tissue RMS did not exhibit a significant correlation with the nose and lip RMS values. However, it was significantly correlated with that of the ramus in both MA and SA groups. Additionally, the soft-tissue chin RMS also showed significant correlation with the ramus and corpus RMS in the SA group.

## Discussion

Facial symmetry is generally believed to be more attractive and is thought to be an important signal of good health and developmental stability [2, 26]. Although asymmetry is a normal biological phenomenon, it may exert a significant influence on facial appearance, resulting in poor psychosocial characteristics and, most importantly, affecting the individual’s quality of life, especially in patients with moderate to severe asymmetry [13, 27]. Accordingly, there is an urgent need to analyze the morphological characteristics of subjects with different degrees of asymmetry to provide a basis for diagnosis and treatment planning. In our study, sex differences, sagittal skeletal patterns and the relationship between entire and individual hard and soft tissues were analyzed in subjects with varying degrees of menton deviation from the MSP. This asymmetry was analyzed by using 3D mirroring and colormap quantification, combining CBCT and 3dMD using digital techniques to potentially compensate for the perspectives in providing an accurate representation of asymmetry and further facilitating the development of remedies for facial asymmetry.

Soft tissue asymmetry, which can more directly affect aesthetics than hard tissue, is currently the focus of much greater attention, owing to patients’ desire to improve their aesthetics and body self-concepts [28]. Various 3D techniques have been applied to investigate soft-tissue facial symmetry, including facial casts [29], laser scans [30] and CT [31]. Facial casts have many limitations, including their high time consumption, discomfort, excess use of materials and so forth. Three-dimensional laser scans provide a noncontact approach to study soft tissue that is more accurate than facial casts [32]. However, the 10-second shooting time is too long to avoid capturing brief facial microexpression. Additionally, radiographical images like CT and CBCT are also exerted. However, soft tissue extracted from CT in the supine position is affected by gravity; in addition, soft tissue from CBCT, might cause distortions due to the pressure of the chin cap. Currently, 3D facial photographs are obtained to analyze the soft tissue prior to the use of the other techniques due to the short capture time, comfortableness of the procedure and ability to produce high-resolution color images [33]. In our study, we combined CBCT with 3dMD, a high-precision and noncontact 3D surface imaging technology with a 2 ms capture time, to digitally investigate the deviations of the hard and soft tissues simultaneously [34].

The soft tissue serves as the external manifestation of the underlying osseous structure. Although facial symmetry is primarily determined by skeletal tissue, it is the soft tissue that shape the facial contours and ultimately determine overall facial symmetry, thereby directly affect appearance [35, 36]. For patients with severe facial asymmetry, orthognathic surgery might significantly improve soft tissue symmetry [4]. In our study, correlation was observed among soft- and hard-tissue as well as anatomical regions which is in consistent with the previous study [1]. Additionally, we found that soft tissue exhibited greater symmetry than hard tissue, suggesting that it might compensate for the latter. This phenomenon might be attributed to variations in the soft tissue thickness and the mastication preference [37].

Sex discrepancies in facial asymmetry are a controversial concept. Many researches have suggested that facial asymmetry presents similarly in males and females [38]. However, in the present study, the RMS values of the entire hard- and soft-tissue were significantly different between males and females, with means of 1.308 and 1.053 mm in males and 1.213 and 0.915 mm in females, respectively. Among individual hard-tissue regions, males had higher RMS values than females which is accordance with Mendoza et al. and Saglam [11, 39]. This contradiction might be caused by sample selection bias, investigation method and techniques, as the inclusion criteria and measuring methods are different among the indicated studies and the current study.

In our study, we found that in the MA and SA group the mandible region was significantly different in both sexes, while most regions showed no significant difference between the RS and MA group in males which suggested that when the menton deviation was larger than 4 mm, asymmetry was much more obvious in hard- and soft-tissue in males. Dentition in males rather than females was significantly different in MA and SA group even the compensatory of dentition exists which might because of the severe asymmetry of males compared to females. In females, only the corpus and ramus region was significantly different among three groups suggested that the asymmetry was mainly caused by the deviation of the mandible, which is in accordance with the findings of Thiesen, et al. [21] Considering the larger degree of asymmetry in hard-tissue compared to soft-tissue in both sexes, we suggested that the soft-tissue might compensated the asymmetry to some extent. The highest mean value of the RMS was shown in the zygomatic process in our study. The zygomatic process was extracted through the orbital point in the present study, which may affect the morphology of the zygomatic bone and further overestimated the RMS. In general, this is a limitation of our study. We will determine the influence of the MSP on the symmetry analysis for each anatomical region and extract the zygomatic bone more completely in the future.

Little difference was observed in the various sagittal skeletal classes in our study. In both sexes, no difference was found in the entire hard- and soft-tissue RMS, which is similar to the study of Haraguchi et al. [22]. In males, the dentition asymmetry was more severe in Class II than in Class I. Among Class II malocclusions, 45–50% were observed in patients a Class I relation on one side and a Class II on the other side, clearly expressing an asymmetry in dentition [40]. Additionally, the asymmetry of the corpus was greater in Class II and III than in Class I, while no difference was observed between Class II and III, which is consistent with previous studies that performed analyses with linear measurements [10, 19]. With regard to the soft tissue, in males, the asymmetry of the cheek was higher in Class III than in Class I. Among females, no significant difference was found among Classes I, II and III. This might be due to the less obvious asymmetry among females than males. The findings above assumed that bilateral disharmony is represented in the same way regardless of sagittal skeletal pattern for females and in most regions for males.

To achieve a foundation for reconstruction, orthodontic and orthognathic treatment, it’s of urgent need to analyze the correlation of the hard- and soft-tissue of each segmented structure, especially the relationship between the deviation of the soft tissue and dentition. As orthodontic treatment leads to tooth movement, while orthognathic treatment seeks to rectify abnormal skeletal structures, the choice among the two treatments for patients with RS or MA remains controversial [36]. Lee et al. and Gaddam et al. found that orthodontic treatment can improve the symmetry of soft tissue in RS patients by comparing the proportions of the bilateral soft-tissue areas before and after treatment [41, 42]. In our study, we found the association of hard- and soft-anatomical structures was comprehensive, and the 2 mm deviation of menton seemed to be a suitable threshold for the hard- and soft-tissue relationship. In the RS group, the whole soft tissue was medium correlated with the dentition, while in the MA and SA groups, it had a medium correlation with the ramus and corpus in males. This suggests that in the RS group, orthodontic treatment might greatly improve facial symmetry, while in the MA and SA groups, orthognathic surgery is needed if the patients pursue obvious improvement in facial symmetry. Additionally, because hard tissue symmetry is related to not only the mandible but also the maxilla, it is better to conduct bimaxillary surgery rather than single jaw surgery, which is in accordance with Wermker et al. [43] and Li et al. [44].

The current study has demonstrated that the integration of CBCT and 3dMD can be utilized in clinical examinations to enhance diagnostic accuracy and evaluate treatment efficacy for asymmetry. For patients with menton deviation less than 2mm, orthodontic treatment is recommended to improve facial symmetry; however, for those with greater deviations, orthognathic surgery is advised to achieve optimal symmetry. Additionally, as the maxilla and zygomatic processes are closely related to facial asymmetry, bimaxillary surgery was suggested instead of mandibular surgery alone.

In the present study, several factors, such as sex, sagittal skeletal patterns and anatomic structures, were considered simultaneously. The use of digital registration of the original and mirrored images combining CBCT and 3dMD was suitable to the study of facial symmetry. However, a number of limitations should be noted, including the following: the subjects were only adults; no comparison was made between pre- and post-treatment; the asymmetry of whole face was solely relied on menton deviation; and vertical growth patterns were not investigated. In the future, we will further analyze growth factors affecting symmetry, complete patient follow-up through completion of orthodontic or orthognathic treatment, investigate the influence of MSP on different anatomical regions when analyzing symmetry, select a larger sample size to further analyze facial asymmetry in patients with specific factors like cleft lip and condylar pathology, apply a more comprehensive classification method like asymmetry index, and measure the facial soft-tissue thickness at the same time in cases of variation caused by soft tissue.

## Conclusions

The mirroring method allowed calculation of the deviation after registration using CBCT and 3dMD images and provides a new approach for digital symmetry analysis. First, males were more asymmetrical than females in most structures. Second, the degrees of asymmetry of both the hard and soft-tissue covaried with the deviation of the menton, which suggested that the classification based on the menton point was reasonable. Third, little difference was found among Classes I, II and III, which demonstrated that sagittal skeletal patterns express facial symmetry equally. Fourth, the facial symmetry of patients with a menton deviation no larger than 2 mm might be rectified by orthodontic treatment. When the deviation is larger than 2 mm, orthognathic treatment should be considered. Finally, orthognathic treatment should focus not only on the lower third of the face but also on other regions, such as the maxilla and zygomatic process. In addition, more samples are needed, especially for patients requiring follow-up therapeutic evaluations.

## Supplementary Information

**Additional file 1: Supplementary Table 1.** Descriptive data of different menton deviation in males. **Supplementary Table 2.** Mann-Whitney U test of RMS of different menton deviation in males. **Supplementary Table 3.** Descriptive data of different menton deviation in females. **Supplementary Table 4.** Mann-Whitney U test of RMS of different menton deviation in females. **Supplementary Table 5.** Descriptive data of different skeletal classes in males. **Supplementary Table 6.** Mann-Whitney U test of RMS of different skeletal classes in males. **Supplementary Table 7.** Descriptive data of different skeletal classes in females. **Supplementary Table 8.** Mann-Whitney U test of RMS of different skeletal classes in females. **Supplementary Table 9.** Correlation coefficient values of hard- and soft-tissue in males. **Supplementary Table 10.** Correlation coefficient values of hard- and soft-tissue in females.

## Data Availability

All the dataset that supported the findings were obtained from the Stomatology Hospital of Xi’an Jiaotong University, Xi’an, Shaanxi, China. The data cannot be available publicly without the permission of the hospital, however, the data was available from the corresponding author on reasonable request.

## Abbreviations

RS: Relative symmetry
MA: Moderate asymmetry
SA: Severe asymmetry
MSP: Mid-sagittal plane
RMS: Root mean square
2D: Two-dimensional
3D: Three-dimensional
CBCT: Cone beam computed tomography
